# Writing Blindly in Incomplete Locked-In Syndrome with A Custom-Made Switch-Operated Voice-Scanning Communicator—A Case Report

**DOI:** 10.3390/brainsci12111523

**Published:** 2022-11-10

**Authors:** Marco Caligari, Marica Giardini, Marco Guenzi

**Affiliations:** 1 Integrated Laboratory of Assistive Solutions and Translational Research (LISART), Istituti Clinici Scientifici Maugeri IRCCS, Scientific Institute of Pavia, 27100 Pavia, Italy; 2 Istituti Clinici Scientifici Maugeri IRCCS, Scientific Institute of Veruno, 28010 Gattico-Veruno, Italy

**Keywords:** interaction/communication competence, locked-in syndrome, motor control accessing, single-switch scanning access, speech impairments

## Abstract

Background: Locked-In Syndrome (LIS) is a rare neurological condition in which patients’ ability to move, interact, and communicate is impaired despite their being conscious and awake. After assessing the patient’s needs, we developed a customized device for an LIS patient, as the commercial augmentative and alternative communication (AAC) devices could not be used. Methods: A 51-year-old woman with incomplete LIS for 15 years came to our laboratory seeking a communication tool. After excluding the available AAC devices, a careful evaluation led to the creation of a customized device (hardware + software). Two years later, we assessed the patient’s satisfaction with the device. Results: A switch-operated voice-scanning communicator, which the patient could control by residual movement of her thumb without seeing the computer screen, was implemented, together with postural strategies. The user and her family were generally satisfied with the customized device, with a top rating for its effectiveness: it fit well the patient’s communication needs. Conclusions: Using customized AAC and strategies provides greater opportunities for patients with LIS to resolve their communication problems. Moreover, listening to the patient’s and family’s needs can help increase the AAC’s potential. The presented switch-operated voice-scanning communicator is available for free on request to the authors.

## 1. Introduction

Brain stem injuries caused by stroke, trauma, anoxia, or even neurodegenerative diseases, e.g., amyotrophic lateral sclerosis (ALS), can lead to a state of Locked-In Syndrome (LIS) [1]. The term was first used by Plum and Posner in 1966 [2] to identify a condition in which a person is severely paralyzed but has intact cognitive skills [1]. The syndrome is a consequence of traumatic or vascular damage that bilaterally disconnects the corticospinal and corticobulbar tracts, and it can have different forms. The classification is based on the amount of motor outputs affected. In the total form, no movement can be produced; in the pure form, patients can move their eyes and blink but are unable to produce any other movement; in the incomplete form, some voluntary movements other than with the eyes remain [3]. LIS rarely shows any improvement. The most common form has a chronic pattern [4,5,6].

The major challenge in LIS is communication, which is essential for the quality of life (QOL) [7], emotional state, and sense of existence of the locked-in person. Communication is not only vital for practical daily life activities, but it is also essential in enabling social participation [8]. People with incomplete LIS can also benefit greatly from communication aids, but the traditional augmentative and alternative communication (AAC) devices are not always a solution, particularly if the residual muscle activity is insufficient to control the device [9].

Re-establishing a communication channel for people with LIS has always been a challenge. Starting with low-tech AAC, i.e., non-battery powered methods of communicating that are usually cheaper to make, patients seek to overcome their communication barrier. Some studies have reported the use of Morse code (by eye blinking) or other eye-coded methods by these patients to transmit a message letter by letter to their interlocutor [10]. Another system is partner-assisted scanning (PAS), in which the caregiver (partner) is in front of the subject and verbally “scans” the sequential items to select [11]. Alternatively, the partner can point out the letters on a visual scanning device (i.e., stating “this row” and scanning through each letter). The patient is only required to look up or down, the easiest way to indicate the letter chosen [12].

In addition, there is high-tech AAC, which includes various technologies and devices to restore the ability to communicate, such as ETCDs (eye-tracking computer devices for communication) [13], BCIs (brain-computer interfaces) [14], ERP (event-related potentials)-based BCIs [15], motor imagery BCIs [16], SSVEP (steady-state visual evoked potential) BCIs [15,17,18], hybrid BCIs [18] combining several techniques (e.g., eye-tracking and electrooculography), and electromyographic (EMG) switches [14]. More recently, a new high-tech approach, experimented in a LIS patient, utilized the intracortical signals to select letters and, thus, write words [19]. Although very exciting, this new approach has so far been used in only one case report where it was invasive, requiring a surgical procedure conducted by an ultra-specialized team with a specific instrumentation.

Communicating with AAC requires strategies for the formulation, storage, and retrieval of words, codes, and messages [20]. A wide variety of software options exist for generating messages, e.g., spelling words letter-by-letter, use of symbols or sequence icons to represent words/messages, selecting words from a display to compose a message, or use of programmed messages that can be retrieved. Each option suits some individuals more than others. Thus, careful consideration is required to match the individual with the most appropriate system [21,22,23,24,25,26,27]. Moreover, the management of the message can be done in different ways. Most AAC technologies use aided symbols with visual displays of pictures, alphabet, pictorial symbols, or codes from which the individual selects [20]. For people with visual impairments, AAC technologies can present spoken messages or tactile representation [28].

All in all, it can be difficult to determine the advantages of one method over another, given the complexity of the individual factors involved (e.g., fatigue, medication, pain, alertness, mood) [29]. This can also make it difficult to determine when and if it is appropriate to change methods; to date, there is still no software that can provide dynamic recognition of these factors, so clinicians and caregivers play a fundamental role in spotting, evaluating, and reporting changes in a patient’s needs. Given the unique characteristics of each individual, the available technology might not be able to meet their needs and it may be necessary to modify existing commercial devices [30].

Our study’s participant was in the locked-in state due to a ponto-cerebellar stroke. At the time of the study, she was using a no-tech AAC [31] that allowed her to communicate with her caregivers but in a very energy-expensive way; she had not been using such technology previously. Her family contacted us because she had read about the integrated Laboratory of Assistive Solutions and Translational Research in which physiotherapists study the possibility of using customized AAC methods. Although she could still communicate via eyebrow movements, she wanted to test other methods as an alternative, since she had a slight voluntary control and repeatable movements of the left thumb (that appeared a few months after the stroke). Hence, the aim of our study was to test a high-tech AAC that she could control without the caregiver’s help, as an alternative to her current method. After comparing various AAC interfaces using different modes for signals acquisition, we found that the most effective AAC method for this user was actually the least expensive.

## 2. Material and Methods

### 2.1. Participant

At the time of the study, the patient (a 51-year old Italian woman, under the pseudonym of Lisa) had been in the locked-in state for 15 years. Lisa was paralyzed since the birth of her daughter, due to a ponto-cerebellar hemorrhage that occurred shortly after the birth. The diagnosis of incomplete LIS [3] was made a few weeks after the stroke, when Lisa showed an ability to answer closed questions by elevating her left eyebrow. A few months later, slight voluntary and repeatable movements of the left thumb appeared. The strength of the thumb was assessed with Kendall’s scale [32]: both the flexion and extension movements had a strength of 3/10 (i.e., movement through a moderate execution range). She was able to sit in a wheelchair, but trembled, had difficulty controlling her head, and was not able to control her upper body. She could voluntarily move the left eyebrow and the distal phalange of the left thumb. Her eyes moved only vertically with continuous and uncontrolled movements, not functional for communication. She was fully assisted 24 h a day.

### 2.2. Conventional Communication

Lisa began to communicate entire words and sentences a few months after the vascular injury by slightly twitching her left eyebrow, choosing the desired letters of the alphabet which were spelt out vocally by her caregiver. This acoustic scanning method, though, was highly demanding both for Lisa and the caregiver, without whom this approach would not have been possible. Lisa’s dad began looking for a solution that would improve his daughter’s ability to communicate with others.

From an Internet search, he contacted different professionals—without good feedback—before he found the integrated Laboratory of Assistive Solutions and Translational Research of the Istituti Clinici Scientifici Maugeri and decided to pursue the matter with them immediately. Lisa and her family were invited to the Institute, where they explained the strategy she used for communicating and her desire to find a better method of communication. The laboratory team was enthusiastic about accompanying her and exploring a possible solution; the decision to write this case report was based on Lisa’s satisfaction with the solution found and her written words. Moreover, based on Lisa’s needs, we created a software that today we wish to make available to all free of charge, considering it an intuitive tool, easy to manage, and immediately available, without excessive instrumentation. Therefore, Lisa gave her informed consent to the present report and to give her testimony. All experimental procedures were conducted in accordance with the Declaration of Helsinki, and with ethical approval of the institutional review board.

### 2.3. Procedure

The researchers began by testing some commercial AAC devices that were available in the Laboratory. Obviously, low technology systems (i.e., non-electronic) were a priori discarded.

High-technology systems include voice output communication aids (called “speech-generating devices”), software on personal computers or laptops, tablet touch-screen devices and mobile phones, which provide multiple access strategies, customizable content, and voice output function [33]. However, these mainstream aids with a relatively low cost and high accessibility are not useful for people with severe motor impairments unable to use standard interfaces [34].

Consequently, for Lisa, the first trial we carried out was with an eye-tracking computer device (Mytobii P10 Eye Tracker^1^), designed as an aid to communication for individuals with minimal movement [35]. However, this eye-tracking was immediately discarded because Lisa, like many patients with LIS, had no horizontal eye movements [3] and her vertical movements were strongly disturbed by continuous involuntary movements. She could not point or keep her gaze fixed on a target for more than 0.5 s, which made interaction with the eye tracker impossible. Next, a sensorized headset for BCI (EMOTIV EPOC®^2^) [1] was tried, in search of some useful signal for communication. Continuous involuntary head movements and facial and cranial muscle spasms created high levels of electroencephalogram contamination; as a result, the BCI option was also abandoned.

Then, the decision was made to use a method that conceptually came very close to the communication system that Lisa and her caregiver were currently using: an auditory scanning AAC. BCIs using the auditory steady state response (ASSR) or the auditory P300 for interaction with BCI devices [36] were excluded for the above-mentioned reasons. The conceptually similar commercially available software required a payment, and we were not sure that they were appropriate for Lisa. Therefore, we decided to develop a custom-designed (both hardware and software) scanning communicator, controlled by a switch that Lisa could move with her left thumb to command a computer screen.

### 2.4. Assessment

**Figure 1:**
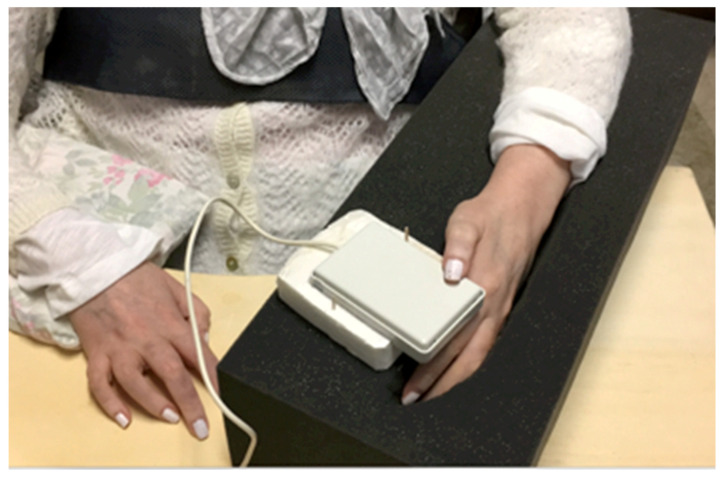
Finding the most suitable arm position for Lisa. In order to satisfy Lisa’s needs, different upper limb positions were tested. Finally, Lisa’s arm and hand were set in a foam pad with the thumb placed on the control switch. Lisa can voluntarily move only her left thumb; the table and the foam allow her to maintain her arms in a comfortable and stable position.

To evaluate Lisa’s effective ability to interact with a switch, a single session of the click-test [37] was administered by a physiotherapist with experience in the AAC field. The test required to click (activate/deactivate) the control switch as many times as possible in 30 s, and then count the number of activations. Lisa was able to click the switch 41 times in the given time, well above the minimum required level of 15 clicks. Because Lisa could not control her hand or arm, the instability of the trunk initially caused many dysfunctional movements of the thumb and provoked loss of contact and unintentional activation of the switch. To solve this problem, the limb was stabilized on a foam pad shaped to accommodate the arm and hand, and the switch used for the click test was fixed in the most suitable position with double-sided tape. All devices and aids, including the laptop, were placed on the wheelchair table for the test (Figure 1).

### 2.5. Custom-Device Development

#### 2.5.1. Hardware

To allow Lisa to interact with the computer, we opted for a mechanical switch, based on a lever system and previously created for patients with ALS [37] (see Appendix A). The switch needed to be both easy to activate voluntarily and at the same time have a certain resistance to unintentional micro-movements that could activate it at the wrong time. This meant positioning the thumb on the lever arm about 80% of the way down distally from the fulcrum.

#### 2.5.2. Software

The initial idea was to utilize the same method as a typical scanning grid communicator, in which the user selects letters and commands highlighted on a screen; however, the main obstacle was to provide information about the real time position of the scanning marker without using the heavily compromised visual channel. Thus, we decided to use the auditory channel (the computer’s speech synthesis) but no software or application for this existed. Therefore, we created a new alphabetical virtual keyboard with a vocal scanning-access, starting from a scanning-access keyboard previously designed for patients with severe motor disabilities. First, letters and commands were grouped into six sets, from “A1” to “A6”. The speech synthesis was programmed to sequentially pronounce the names assigned to each set (“A1, A2, [...], A6”) cyclically, and then each item of the group selected by the user. The message the user wants to transmit gradually appears in a dedicated text box (see Appendix A).

**Figure 2:**
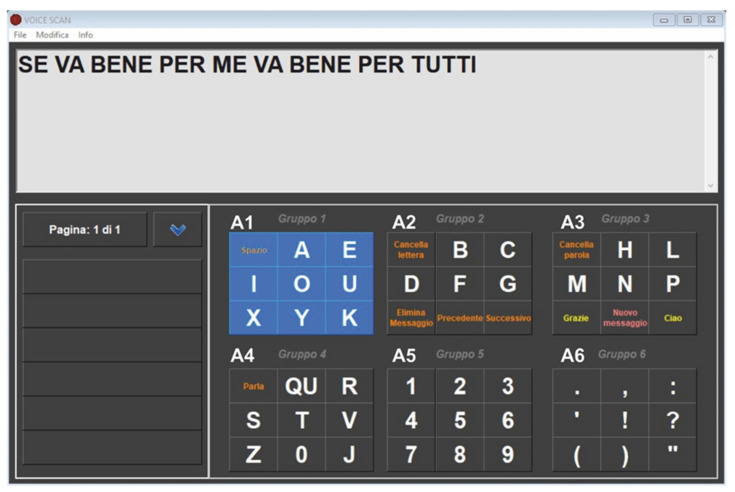
Lisa writes her opinion on her new AAC. The computer writes in response to Lisa’s commands with her left thumb click. Lisa cannot see the scanning marker, she can only hear the corresponding code: “A1” for group 1 (‘gruppo’ means group in Italian), “A2” for group 2, and so on. After the first click (group selection) by pressing the control switch, she hears the letters of the selected set, spelt out one after another (e.g., A-E-I-O-U-J-X-Y). A second click chooses the desired letter. This screenshot shows one of the first sentences written by Lisa (translated from Italian “If it works for me, it works for everyone”), who motivated this present article.

Figure 2 shows the dashboard of the software, written in Italian, Lisa’s language. Vowels were positioned in the first set followed by consonants, generally respecting alphabetical order to improve the software’s usability and make it easier for the user to remember the position of each letter. For the same reason, the more uncommon Italian letters were placed at the end of the sets, to avoid spelling them out in vain. Moreover, in accordance with Lisa’s preferences, commands were placed in various sets (i.e., “space” in A1, “speak” in A4, etc.). A practice trial is presented in video-format in Supplementary Materials.

## 3. Results

The device was run, and Lisa listened to the synthetic voice describing the content of each group for 2 min, so as to make a mental map of the position of each letter or command. As a first trial, in order to verify that the system as a whole worked properly, we asked her to write her name and those of her children and she did so immediately. Then we asked her what she thought about the device overall. She typed, in about 3 min, without making any typing mistakes: “Se funziona per me funziona per tutti” (If it works for me, it works for everyone) (Figure 2). While her answers were being video-documented, Lisa typed, on her own initiative “Può andare più veloce?” (Can it go any faster?), referring to the advancement speed of the scanning marker. Through a trial-and-error process using different timings, we were able to reduce the scanning pause from 1200 to 800 milliseconds; with this speed, Lisa could comfortably type letters, without too-long a latency time. The new setting did not result in any typing errors, and Lisa confirmed that this was the setting she preferred.

### Effectiveness of the Custom Device

Two years after development of the switch-based communicator, Lisa returned to the laboratory for a follow-up regarding use of the device. She reported that she was able to use the device for just over ½ an hour at a time, after which she found it tiring. She used the communicator 2–3 days a week on average, not every day due to problems mostly related to health or assistance. The communicator was usually placed on a table with wheels so it could be easily moved and used both when in bed and in her wheelchair. During her daily routine, the time required for the caregiver to position Lisa’s arm on the foam pad, her thumb on the control switch, and set up the device for use was approximately 2–3 min. The scanning pause Lisa used was 800 milliseconds, and she was able to write over 2 words per min: one character every 4.55 s on average. She generally wrote messages to those who assisted her, especially her family members and children. Even though the time taken was slightly longer than with the previous communication modality, she could write by herself and choose her words without suggestions, i.e., she was more autonomous.

In order to determine the overall usability of the device, including Lisa’s confidence with it for daily living activities, we asked Lisa and her father (the main caregiver) to complete the following questionnaires:

- Global Rating of Change scale (GRC). This single-item scale assesses the perceived improvement in quality of life with use of the new device. The subject grades the perceived improvement (or lack of improvement) on a 15-point scale from −7 (lack of improvement) to +7 (excellent improvement) [38].
- Quebec User Evaluation of Satisfaction with Assistive Technology (QUEST 2.0) questionnaire. This 12-item scale assesses user satisfaction with an assistive technology device. The scale investigates two dimensions: satisfaction with the device and satisfaction with the service. Item scores range from 1 (not satisfied at all) to 5 (very satisfied) [39]. Only the eight items related to satisfaction with the device (QUEST 2.0-Dev) were reported for this study.

In the GCR, Lisa’s rating on how much the communicator had changed her quality of life was 3, while her father rated it 5. Regarding satisfaction with the communicator, Table 1 reports the results on QUEST 2.0. Even if other future improvements might be possible, Lisa and her father were generally satisfied about the characteristics of the customized device. In particular, Lisa gave the top score (5) for the effectiveness of the device; she found it well suited to her communication needs.

## 4. Discussion

Facilitating communication for individuals with severe motor impairments such as LIS can be extremely difficult either because an access solution is unavailable or it is unreliable, causing frustration and limited use of the technology [40]. When both vertical and horizontal ocular movements are present (e.g., in many cases of advanced-stage ALS), AAC devices using gaze pointing should be used to recover communication [3] and to improve life quality. More often, in traumatic or vascular Locked-In Syndrome, BCIs can provide a muscle-independent communication channel [41]. In 1988, researchers proposed a BCI based on ERPs in the electroencephalogram (EEG) such as P300 [42]. Today, many kinds of BCIs exist, such as motor imagery BCIs [16], SSVEP BCIs [15,17,18,43], or Hybrid BCIs [18] that conjugate several techniques (such as eye tracking and electrooculography) with the aim of improving reliability and speed in human-machine interaction. BCI techniques first need to acquire EEG signals from the user’s brain. The most non-invasive methods of EEG detection use helmets or a headset with dry or wet surface electrodes placed on the scalp [44,45]. The acquisition of EEG signals is a critical operation that can be affected by disturbances and artefacts [46]. EEG contamination from the cranial muscles is often present in the early stages of BCI training but it gradually wanes [14]. Some users never acquire EEG control. In these cases, electromyography (EMG) may be used to move the cursor toward the target [14]; for example, the EMG signal can be used as a trigger to control a scanning communicator with an alphabetical onscreen keyboard [47] or to select virtual directional arrows that move the cursor across the screen [43]. However, dystonic movements, eye blinking, clenching of teeth, or continuous facial or cranial muscle spasms lead to a high rate of EMG contamination [48], which represents the main cause of failure [14]. This situation occurred in our case: due to continuous artefacts, Lisa would not have been able to use either a BCI-based communication system or the EMG signal acquired from the scalp as a trigger to control a scanning communication system.

Some studies [10] have reported use of the Morse code or other eye-coded methods by LIS patients to transmit a message letter by letter to the interlocutor, even if the patient’s eye movements may be inconsistent, very small, and easily exhausted [49]. In 1997, Jean-Dominique Bauby wrote an entire (and successful) book, “The diving bell and the butterfly”, dictating it to a typist only by eye blink [50]. A study conducted in 2012 described a patient with LIS who was able to communicate, after 3 weeks of training, through a virtual scanning keyboard thanks to detection of the eye blink by an infrared sensor mounted on a pair of glasses [51]. This method requires the ability to observe the screen, follow the progress of the scanning cursor over the items, and wink with precision at the right time—skills that are not always available in patients with LIS. In another recent study, an electrooculogram-based auditory communication system was used by a small group of patients with incomplete LIS [52]. They had no gait fixation but were able to partially control the eye movements. Nevertheless, only 1 patient had sufficiently good oculomotor control, as reflected in electrooculogram signals, to use this AAC aid. Therefore, the biggest issue is the fact that each patient has a unique clinical picture, with distinct abilities and disabilities; Lisa could in no way use her eyes to communicate.

The multiplicity of clinical manifestations makes it difficult to find an easy, standard solution to help these patients interact with their environment and to lay the foundations for further research to find ways to suit their varied needs. This is what we accomplished for Lisa by creating a customized device specifically for her, but which could also serve other patients with LIS with similar characteristics.

First, we created a software program with a specific application describing the progress of the scanning marker with speech feedback. Then, we discussed and defined some issues for the practical implementation of the scanning-based communicator. We considered whether it was better to adopt an automatic scanning engine (requiring only one user movement to select the desired item) or a manual one (faster but requiring more than one switch and many clicks for one selection). Then, we had to choose the hardware, in particular the control switch. There are many switches on the market with different activation modes: mechanical, capacitive, electromyographic, optical, sound, blowing, or suctioning. It was necessary to identify which part of the body to use to interact with the switch, which kind of button to use, how to stabilize it, and how to posture the patient to ensure comfort and minimize fatigue.

Furthermore, in order to use an alphabetical communication device with scanning access, the patient must have adequate cognitive skills, together with very good mnemonic abilities. In addition, the capacity to interact with the command switch with a certain reactivity and reliability is required; otherwise, the user might press the switch when the scanning marker has already moved on to the next item. In our intervention, the click test confirmed that Lisa was able to control the switch. Moreover, among the first written sentences Lisa declared “If it works for me, it works for everyone” (translated from Italian); this remark appeared as an invitation to spread her story to give other people in the same condition a new possibility for communicating.

## 5. Conclusions

The intervention described in this case study was successful for several reasons. First, it is thanks to Lisa’s excellent intact cognitive skills that she is able to write and communicate with the world around her. Indeed, she has no difficulty in understanding; thanks to her 10-year long experience in vocal scanning-writing with the caregiver, she was able to easily start using the computer vocal scanning. Moreover, Lisa has a good memory and learnt the letter distribution in each box without problems.

Second, it is thanks to her father, who did not give up the search for a solution, as well as to her family and friends, who organized for Lisa the most suitable conditions in which to use the communicator. Finally, the fact of being able to modify the whole device—both its hardware and software components—enabled us to create a solution that was well suited to Lisa’s needs.

Another reason for the overall success was the close involvement of Lisa and her father in the development of the device. We believe that it is very important to involve users themselves in the development process in order to customize the device to their needs. For example, Lisa asked us to speed up the scanning marker and then to insert recurring courtesy phrases in the communicator interface (“Thanks”, “Please”, “I love you”, and others). The laboratory team would not have been able to conceive this sort of detail; only a real user can suggest such improvements to optimize the result personally. Moreover, after using the device for about 1 month, Lisa requested that Arabic numerals be added and that the layout of the keyboard be modified, moving some letters and commands from one position to another so as to speed up the writing process and make use easier. The fact that she is still using the device after over 2 years is a sign that it is technically reliable and sufficiently functional and effective to meet her communication needs, an assumption confirmed by our follow-up assessment.

The limitation of our device appears to lie in the cognitive load it requires and produces: Lisa can use the communicator for only about ½ an hour at a time, after which she gets tired and has to stop. However, practice and habit might improve ability in using the device and so reduce fatigue, decreasing the cognitive load. It seems clear that a good cognitive level is crucial to access this communicative channel, as it is demanding on time, attention, and concentration. In fact, patients who have significant linguistic and cognitive limitations (e.g., persons with dementia) often have difficulty learning to use traditional AAC technologies [53]. Moreover, there is no validated evaluation to assess the psycho-physiological states of patients with LIS [54].

In our case study, the tests that we carried out with the most common communication systems and facilitated access methods (eye tracker, BCIs, scanning with visual feedback) failed. This implies that, in the field of AAC, one cannot rely on standard solutions despite the advantages that each AAC method may offer. For instance, Lisa’s complex needs (the LIS condition coupled with her specific involuntary eye movements, and trembling) ruled out interaction with BCI or eye-tracking computer devices. We should point out that we did not test all AAC devices on the market, for budget reasons (both ours and Lisa’s): the more sophisticated the instrument, the higher the cost. In our clinical setting, we did not have at our disposal the full range of commercial devices available on the market. However, even if it were possible for our Institute to purchase a specific tool, the same would not be true for a “normal” family. Thus, we preferred to develop a custom-made tool, which was very cheap and fitted well Lisa’s needs.

We agree that there is need for continuing research to develop new alternative or facilitated access methods and provide more customized AAC devices. The design process we followed was person-centered, in line with theoretical frameworks for medical device and assistive technology development [55]. The challenge is to ensure that all individuals have access to effective means of communication and can participate in social relations to the fullest extent possible.

Finally, if the reader is interested in testing and using our AAC tool, please contact the authors. We will be pleased to share free of charge this software, as our contribution to the field.

## Figures and Tables

**Table 1:** Quebec User Evaluation of Satisfaction with Assistive Technology.

Assistive Device		Lisa	Father
1.	How satisfied are you with the dimensions (size, height, length, width) of your assistive device?	5	4
2.	How satisfied are you with the weight of your assistive device?	NA	5
3.	How satisfied are you with the ease in adjusting (fixing, fastening) the parts of your assistive device?	3	3
4.	How safe and secure your assistive device is?	4	4
5.	How satisfied are you with the durability (endurance, resistance to wear) of your assistive device?	5	5
6.	How easy it is to use your assistive device?	4	NA
7.	How comfortable your assistive device is?	3	NA
8.	How effective your assistive device is (the degree to which your device meets your needs)?	5	NA

Item scores are: 1 = not satisfied at all, 2 = not very satisfied, 3 = more or less satisfied, 4 = quite satisfied, 5 = very satisfied. NA (non-applicable) was used for items in which Lisa or her father could not answer (i.e., Lisa regarding the weight of the device).

**Figure A1:**
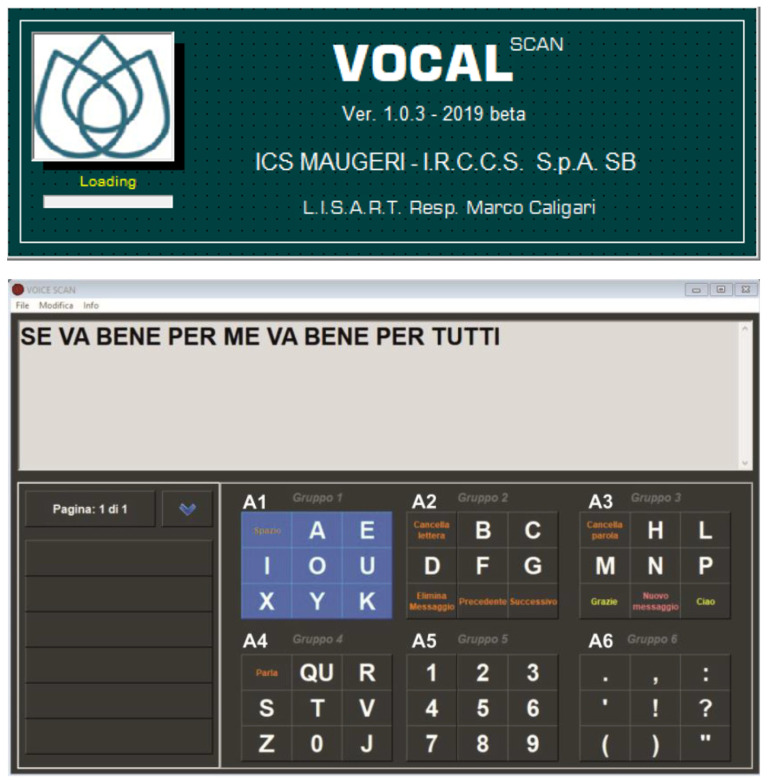
Screenshot showing the text composed by the user. Mytobii© P10 Eye Tracker, Tobii Technology, Danderyd, Sweden.

**Figure A2:**
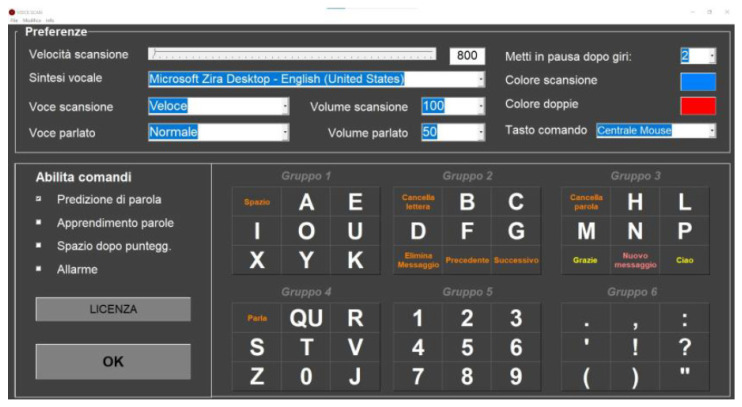
Screen with the program settings. EMOTIV EPOC® 14 channels EEG headset. EMOTIV® San Francisco, U.S.A. https://www.emotiv.com/epoc/.

## Supplementary Materials

The following supporting information can be downloaded at: https://www.mdpi.com/article/10.3390/brainsci12111523/s1.

## Appendix A. Vocal-Scan Software and Hardware Details

### Appendix A.1. Software

VOCAL-Scan (M-VOCAL.exe, version 1.1, Italy) is a AAC application for Windows, developed in VB6, designed for writing by a switch-operated voice-scanning communicator—it is intended for users suffering from severe motor disabilities who have visual problems. The interaction between user and communicator takes place through a switch command. The user clicks it to validate the scanned items, and so is able to compose messages letter by letter without having to see them on the screen.

The app has several options that can be adapted to the user’s needs: (1) adjustable scan progress speed from 400 to 4000 ms; (2) speech synthesis in various languages (Microsoft localized according to the user’s country is the default, but others can be selected); (3) volume and speed of pronunciation of the elements scanned; (4) speed and volume of speech synthesis that reads messages. It is also possible to turn word prediction on/off or automatically enter a space after typing a punctuation character.

Acoustic feedback: As the scan advances, a synthetic voice verbalizes the process. It initially calls the groups with a progressive and cyclic number (1, 2, 3...); after the user has selected the desired group with a click, it verbalizes in succession the elements in the chosen group. The user, on hearing the desired letter (or command), clicks a second time. The voice repeats the item to inform the user of the choice. When the user has written an entire word and typed in a space, the voice repeats it so that the user can verify the correctness of what is written. In the case of error, the user can correct it through the appropriate commands (e.g., delete last letter, delete last word).

### Appendix A.2. Hardware

The app runs on multimedia PCs from Windows 7 onwards, requires a USB 2.1 port or later to power a sensor interface to connect the control sensor, such as the one we use (Gradual).

The software is very lightweight (only 844 Kb) so it does not require any special specifications, although we recommend a PC with a good audio section to provide adequate acoustic feedback to the user.

## References

[1] Käthner I., Kübler A., Halder S. (2015). Comparison of eye tracking, electrooculography and an auditory braincomputer interface for binary communication: A case study with a participant in the locked-in state. JNER.

[2] Posner J.B., Saper C.B., Schiff N. (2007). Plum and Posner’s Diagnosis of Stupor and Coma.

[3] Bauer G., Gerstenbrand F., Rumpl E. (1979). Varieties of the locked-in syndrome. J. Neurol..

[4] Feldman M.H. (1971). Physiological observations in a chronic case of “locked-in” syndrome. Neurology.

[5] Nordgren R.E., Markesbery W.R., Fukuda K., Reeves A.G. (1971). Seven cases of cerebromedullospinal disconnection: The “locked-in” syndrome. Neurology.

[6] Markand O.N., Dyken M.L. (1976). Sleep abnormalities in patients with brain stem lesions. Neurology.

[7] Vansteensel M.J., Jarosiewicz B. (2020). Brain-computer interfaces for communication. Handbook of Clinical Neurology.

[8] Vidal F. (2020). Phenomenology of the locked-in syndrome: An overview and some suggestions. Neuroethics.

[9] Kim D.Y., Han C.H., Im C.H. (2018). Development of an electrooculogram-based human-computer interface using involuntary eye movement by spatially rotating sound for communication of locked-in patients. Sci. Rep..

[10] Kuzma-Kozakiewicz M., Andersen P.M., Ciecwierska K., Vázquez C., Helczyk O., Loose M., Uttner I., Ludolph A.C., Lulé D. (2019). An observational study on quality of life and preferences to sustain life in locked-in state. Neurology.

[11] Beukelman D.R., Light J.C. (2020). Augmentative & Alternative Communication: Supporting Children and Adults with Complex Communication Needs.

[12] Davis T.J. (2020). Auditory Motion Perception: Investigation of Benefit in Multi-Talker Environments. Ph.D. Thesis.

[13] Caligari M., Godi M., Guglielmetti S., Franchignoni F., Nardone (2013). A. Eye tracking communication devices in amyotrophic lateral sclerosis: Impact on disability and quality of life. Amyotroph. Lateral Scler. Front. Lobar Degener..

[14] McFarland D.J., Sarnacki W.A., Vaughan T.M., Wolpaw J.R. (2005). Brain-computer interface (BCI) operation: Signal and noise during early training sessions. Clin. Neurophysiol. Pract..

[15] Maggi L., Parini S., Piccini L., Panfili G., Andreoni G. A four command BCI system based on the SSVEP protocol. Proceedings of the2006 International Conference of the IEEE Engineering in Medicine and Biology Society.

[16] Wierzgała P., Zapała D., Wojcik G.M., Masiak J. (2018). Most popular signal processing methods in motor-imagery BCI: A review and meta-analysis. Front. Neuroinform..

[17] Parini S., Maggi L., Turconi A.C., Andreoni G. (2009). A robust and self-paced BCI system based on a four class SSVEP paradigm: Algorithms and protocols for a high-transfer-rate direct brain communication. Comput. Intell. Neurosci..

[18] Jalilpour S., Sardouie S.H., Mijani A. (2020). A novel hybrid BCI speller based on RSVP and SSVEP paradigm. Comput. Methods Programs Biomed..

[19] Chaudhary U., Vlachos I., Zimmermann J.B., Espinosa A., Tonin A., Jaramillo-Gonzalez A., Khalili-Ardali M., Topka H., Lehmberg J., Friehs G.M. (2022). Spelling interface using intracortical signals in a completely locked-in patient enabled via auditory neurofeedback training. Nat. Commun..

[20] Beukelman D.R., Mirenda P. (2013). Augmentative & Alternative Communication: Supporting Children and Adults with Complex Communication Needs.

[21] Ratcliff A. (1994). Comparison of relative demands implicated in direct selection and scanning: Considerations from normal children. AAC.

[22] Mizuko M., Reichle J., Ratcliff A., Esser J. (1994). Effects of selection techniques and array sizes on short-term visual memory. AAC.

[23] Rowland C., Schweigert P.D., Light J.C., Light J.C., Beukelman D.R., Reichle J. (2003). Cognitive skills and AAC. Communicative Competence for Individuals who Use AAC: From Research to Effective Practice.

[24] Wagner B., Jackson H.M. (2006). Developmental memory capacity resources of typical children retrieving picture communication symbols using direct selection and visual linear scanning with fixed communication displays. J. Speech Lang. Hear. Res..

[25] Higginbotham D.J., Shane H., Russell S., Caves K. (2007). Access to AAC: Present, past, and future. AAC.

[26] Light J.C., McNaughton D. (2013). Putting people first: Re-thinking the role of technology in augmentative and alternative communication intervention. AAC.

[27] Thistle J.J., Wilkinson K.M. (2013). Working memory demands of aided augmentative and alternative communication for individuals with developmental disabilities. AAC.

[28] Flaubert J.L., Spicer C.M., Jette A.M., National Academies of Sciences, Engineering, and Medicine (2017). Augmentative and Alternative Communication and Voice Products and Technologies. The Promise of Assistive Technology to Enhance Activity and Work Participation.

[29] McLaughlin D., Peters B., McInturf K., Eddy B., Kinsella M., Mooney A., Deibert T., Montgomery K., Fried-Oken M. (2021). Decision-Making for Access to AAC Technologies in Late Stage ALS. Augment. Altern. Commun..

[30] Villalobos A.E.L., Giusiano S., Musso L., de’Sperati C., Riberi A., Spalek P., Calvo A., Moglia C., Roatta S. (2021). When assistive eye tracking fails: Communicating with a brainstem-stroke patient through the pupillary accommodative response—A case study. Biomed. Signal Processing Control..

[31] Elsahar Y., Hu S., Bouazza-Marouf K., Kerr D., Mansor A. (2019). Augmentative and alternative communication (AAC) advances: A review of configurations for individuals with a speech disability. Sensors.

[32] Kendall F.P., Provance P.G., McCreary E.K., Crosby R.W. (2000). I Muscoli: Funzioni e Test Con Postura e Dolore.

[33] Ganz J.B., Morin K.L., Foster M.J., Vannest K.J., Genç Tosun D., Gregori E.V., Gerow S.L. (2017). High-technology augmentative and alternative communication for individuals with intellectual and developmental disabilities and complex communication needs: A meta-analysis. AAC.

[34] Mandak K., Light J., Brittlebank-Douglas S. (2021). Exploration of multimodal alternative access for individuals with severe motor impairments: Proof of concept. Assist. Technol..

[35] Ball L.J., Nordness A.S., Fager S.K., Kersch K., Mohr B., Pattee G.L., Beukelman D.R. (2010). Eye gaze access of AAC technology for people with amyotrophic lateral sclerosis. J. Med. Speech-Lang. Pathol..

[36] Brumberg J.S., Pitt K.M., Mantie-Kozlowski A., Burnison J.D. (2018). Brain-Computer Interfaces for Augmentative and Alternative Communication: A Tutorial. Am. J. Speech-Lang. Pathol..

[37] Caligari M., Godi M., Giardini M., Colombo R. (2019). Development of a new high sensitivity mechanical switch for augmentative and alternative communication access in people with amyotrophic lateral sclerosis. JNER.

[38] Kamper S.J., Maher C.G., Mackay G. (2009). Global rating of change scales: A review of strengths and weaknesses and considerations for design. J. Man. Manip. Ther..

[39] Demers L., Weiss-Lambrou R., Ska B. (2002). The Quebec User Evaluation of Satisfaction with Assistive Technology (QUEST 2.0): An overview and recent progress. Technol. Disabil..

[40] Koch Fager S., Fried-Oken M., Jakobs T., Beukelman D.R. (2019). New and emerging access technologies for adults with complex communication needs and severe motor impairments: State of the science. AAC.

[41] Fager S., Beukelman D.R., Fried-Oken M., Jakobs T., Baker J. (2012). Access interface strategies. Assist. Technol..

[42] Farwell L.A., Donchin E. (1988). Talking off the top of your head: Toward a mental prosthesis utilizing event-related brain potentials. Electroencephalogr. Clin. Neurophysiol..

[43] Kapgate D., Kalbande D., Shrawankar U. (2020). An optimized facial stimuli paradigm for hybrid SSVEP+ P300 brain computer interface. Cogn. Syst. Res..

[44] Kim J., Lee J., Han C., Park K. (2019). An instant donning multi-channel EEG headset (with comb-shaped dry electrodes) and BCI applications. Sensors.

[45] Ko L.W., Chang Y., Wu P.L., Tzou H.A., Chen S.F., Tang S.C., Yeh C.-L., Chen Y.-J. (2019). Development of a smart helmet for strategical BCI applications. Sensors.

[46] Usakli A.B. (2010). Improvement of EEG signal acquisition: An electrical aspect for state of the art of front end. Comput. Intell. Neurosci..

[47] Cler M.J., Nieto-Castañón A., Guenther F.H., Fager S.K., Stepp C.E. (2016). Surface electromyographic control of a novel phonemic interface for speech synthesis. AAC.

[48] Lee D., Lee S., Lee K.J., Lee G. (2019). Biological surface electromyographic switch and necklace-type button switch control as an augmentative and alternative communication input device: A feasibility study. Phys. Eng. Sci. Med..

[49] Laureys S., Pellas F., Van Eeckhout P., Ghorbel S., Schnakers C., Perrin F., Berré J., Faymonville M.-E., Pantke K.-H., Dama F. (2005). The locked-in syndrome: What is it like to be conscious but paralyzed and voiceless?. Prog. Brain Res..

[50] Bauby J.D., Leggatt T.J. (1997). The Diving Bell and the Butterfly [Le Scaphandre et le Papillon].

[51] Park S.W., Yim Y.L., Yi S.H., Kim H.Y., Jung S.M. (2012). Augmentative and alternative communication training using eye blink switch for locked-in syndrome patient. Ann. Rehabil. Med..

[52] Tonin A., Jaramillo-Gonzalez A., Rana A., Khalili-Ardali M., Birbaumer N., Chaudhary U. (2020). Auditory electrooculogram-based communication system for ALS patients in transition from locked-in to complete locked-in state. Sci. Rep..

[53] Light J., McNaughton D., Beukelman D., Fager S.K., Fried-Oken M., Jakobs T., Jakobs E. (2019). Challenges and opportunities in augmentative and alternative communication: Research and technology development to enhance communication and participation for individuals with complex communication needs. AAC.

[54] Khalili-Ardali M., Wu S., Tonin A., Birbaumer N., Chaudhary U. (2021). Neurophysiological aspects of the completely locked-in syndrome in patients with advanced amyotrophic lateral sclerosis. Clin. Neurophysiol..

[55] Shah S.G.S., Robinson I., AlShawi S. (2009). Developing medical device technologies from users’ perspectives: A theoretical framework for involving users in the development process. Int. J. Technol. Assess. Health Care.

